# Fitness cost in field and laboratory *Aedes aegypti* populations associated with resistance to the insecticide temephos

**DOI:** 10.1186/s13071-015-1276-5

**Published:** 2015-12-30

**Authors:** Diego Felipe Araujo Diniz, Maria Alice Varjal de Melo-Santos, Eloína Maria de Mendonça Santos, Eduardo Barbosa Beserra, Elisama Helvecio, Danilo de Carvalho-Leandro, Bianka Santana dos Santos, Vera Lúcia de Menezes Lima, Constância Flávia Junqueira Ayres

**Affiliations:** Department of Entomology, Aggeu Magalhães Research Center (Centro de Pesquisas Aggeu Magalhães - CPqAM) – Oswaldo Cruz Foundation (Fundação Oswaldo Cruz - Fiocruz), Recife, Brazil; Department of Biology, State University of Paraíba (Universidade Estadual da Paraíba - UEPB), Campina Grande, Brazil; Laboratory of Lipids, Biochemistry Department, Federal University of Pernambuco (Universidade Federal de Pernambuco - UFPE), Recife, Brazil

**Keywords:** Culicidae, insecticide resistance, organophosphate, temephos, trade-offs, fitness

## Abstract

**Background:**

The continued use of chemical insecticides in the context of the National Program of Dengue Control in Brazil has generated a high selective pressure on the natural populations of *Aedes aegypti,* leading to their resistance to these compounds in the field. Fitness costs have been described as adaptive consequences of resistance. This study evaluated the biological and reproductive performance of *A. aegypti* strains and a field population resistant to temephos, the main larvicide used for controlling mosquitoes.

**Methods:**

Comparative tests were performed with a resistant field population from the municipality of Arcoverde, Pernambuco State, Brazil, with a high rate of temephos resistance (RR = 226.6) and three isogenetic laboratory strains from the same origin (Araripina municipality, Pernambuco): RecR (RR = 283.6); RecRNEx (RR = 250.5), a strain under a process of resistance reversion; and RecRev (RR = 2.32), a reversed susceptible strain used as an experimental control.

**Results:**

Our study revealed that the absence of selective pressure imposed by exposure to temephos, for five consecutive generations, led to a discrete reduction of the resistance ratio and the response of the detoxifying enzymes. Most of the 19 biological parameters were impaired in the resistant strains and field population. The analysis of the fertility life table confirmed the presence of reproductive disadvantages for the resistant individuals. Similarly, the longevity, body size, and total energetic resources were also lower for the resistant females, except for the last two parameters in the field females (Arcoverde). In contrast, the sex ratio and embryonic viability suffered no interference in all strains or population evaluated, regardless of their status of resistance to temephos.

**Conclusions:**

The reproductive potential and survival of the resistant individuals were compromised. The parameters most affected were the larval development time, fecundity, net reproduction rate, and the generational doubling time. These fitness costs in the natural population and laboratory strains investigated are likely associated with maintaining the metabolic mechanism of resistance to temephos. Our results show that despite these costs, the highly temephos resistant populations can compensate for these losses and successfully overcome the control actions that are based on the use of chemical insecticides.

## Background

*Aedes aegypti* is a species of wide geographic distribution that has great epidemiological importance because females of this species can carry several arbovirus, such as Dengue, Yellow Fever and Chikungunya [1, 2]. Due to the absence of a polyvalent vaccine for human immunization against different serotypes of Dengue virus (DENV), vector control through the use of chemical insecticides remains the primary strategy to contain outbreaks of the disease [3]. The intensive and extensive use of the organophosphate temephos for controlling *A. aegypti* worldwide has generated a high selective pressure on mosquito populations, causing changes in the susceptibility of natural populations of this species [4–12], including in Africa [13].

Resistance is a pre-adaptive process resulting from random genetic mutations [3]. Resistance to organophosphates such as temephos may occur due to changes in the target site of the insecticide, which, in this case, is the enzyme acetylcholinesterase, a neurotransmitter present in nerve synapses, or due to accelerated metabolism of the insecticide, which prevents the insecticide from reaching its target [14]. Accelerated insecticide metabolism is caused by the over expression of detoxifying enzymes or enzyme structural changes that increase their metabolic capacity [3]. Until now, no case of natural *A. aegypti* populations with mutations in the enzyme acetylcholinesterase, leading to resistance, has ever been recorded, although it has been described in other diptera [15–17]. Thus, it is believed that metabolic resistance is the process most likely involved in the resistance to temephos in this species [8, 18].

One of the most discussed issues in biological studies involving resistance is the nature of the adaptive process, which, despite often being associated with a fitness cost, leads to the survival and reproductive success of individuals exposed to a natural or induced adverse condition [19–21]. Fitness cost is an energetic investment that leads to incremental losses of biotic potential. Previous work on this subject was performed on *Culex pipiens* (a model organism for fitness cost studies), and the fitness costs have been described as a consequence of the vector/parasite interaction or of the resistance to chemical insecticides [22]. Moreover, with respect to this species, it has been found that genes that confer resistance to chemical insecticides typically carry a number of associated biological costs, such as vulnerability to predation, reduced competitive potential among males, increased development time, decreased size of individuals, and reduced survival rates [23–26].

The amount of some energetic reserves in *C. pipiens* mosquitoes resistant to organophosphates can be reduced due to an over expression of enzymes (esterases) involved in the metabolic process of insecticide detoxification [22]. According to Rivero et al., this reduction is a consequence of metabolic exchange, which is defined by the expression “trade-off”, which means compensation, representing a reallocation of energetic resources from a primary function (such as egg production) to maintain a secondary function (for example, overproduction of detoxifying enzymes) [22]. Thus, resistant insects may have a large adaptive advantage in an environment where there is continuous pressure due to insecticide use [27]. However, their survival in these conditions may represent the reduced performance of certain physiological processes, such as fecundity and longevity of individuals in the field [21].

Thereby, genotypes that confer resistance to xenobiotics may have some adaptive disadvantages compared with the genotypes of susceptible individuals in the absence of the selective pressure exerted by these compounds [27]. Resistance, in most cases, is not stable and tends to decrease significantly over time in the field when the contact with the insecticide ceases. This suggests the existence of a fitness cost related to maintaining the biological mechanisms that provide resistance to insecticides [8].

In the present study, we tested the hypothesis that biological parameters are impaired due to high levels of resistance to temephos (RR > 200) in the *A. aegypti* field population and laboratory strains that are harboring the metabolic resistance mechanism.

## Methods

### Establishment and maintenance of *Aedes aegypti* strains and field population

To perform the experiments in this work we first established the laboratory strains and the field population under controlled conditions in the insectary of Aggeu Magalhães Research Center (Centro de Pesquisas Aggeu Magalhães - CPqAM), Oswaldo Cruz Foundation (Fundação Oswaldo Cruz - Fiocruz). In order to standardize the conditions for rearing larvae, 200 first instar larvae were placed in plastic containers containing 2 L of water and 1 mg of cat food (Friskies®)/per larva for rearing and nine containers for each laboratory strain or population were used, totaling 1800 larvae per group. In the pupal stage, individuals were transferred to containment cages for the emergence of mosquitoes. Males and females were fed a 10 % sugar solution *ad libitum*, and additionally, females were offered four blood meals from Swiss mice (*Mus musculus*) once a week to obtain progenies. Ethical approval: the use of these mice was authorized by the Animal Ethics Committee of the CPqAM, approval no. 27/2011. The female mosquitoes laid their eggs on substrates (filter paper) moistened with water. The moist eggs were partially dried after embryogenesis at room temperature and then stored until use. All the insects were kept in climatized rooms at 26 °C ± 1 °C with a relative humidity of 50 % to 60 % and a 12 h photophase.

### *Aedes aegypti* laboratory strains and field population

Three strains of *A. aegypti* with the same genetic background and a field population from the municipality of Arcoverde, Pernambuco State, Brazil, used in this study, are described below.

1. Recife-Resistant (RecR): The strain, which originated from a field population collected in the municipality of Araripina (7°34′34″ S and 40°29′54″ W), 690 km from Recife, capital of Pernambuco State, has been subjected to high selective pressure with the organophosphate temephos. The RecR strain has been maintained in the Insectary of Department of Entomology of the CPqAM/FIOCRUZ-Pernambuco since 2004 [8]. All the tests in this study were performed with the 26^th^ generation of this strain, with a resistance ratio to temephos higher than >200 fold.
2. Sub-strains Rec-Reverse (RecRev) and RecR Non-Exposed (RecRNEx): the first sub-strain subjected to the process of reversion of resistance to temephos, RecRev, was established from the 14^th^ generation of RecR, when it presented a resistance ratio of 125-fold [8]. The 21^st^generation of RecRev used in this study was considered susceptible to temephos because it presents a resistance ratio <3 fold and exhibits patterns of detoxification enzyme activity similar to the Rockefeller strain (standard susceptible strain). RecRev has been kept without exposure to temephos, and it was used as a control for biological performance of the resistant individuals. The second sub-strain, RecRNEx, was established from the 26^th^ generation of RecR. Selective pressure with temephos was suspended for five consecutive generations to evaluate the biological parameters.
3. Rockefeller strain: This standard susceptibility strain to chemical insecticides, was used exclusively as a control to estimate the resistance ratio and detoxification enzyme activity. This strain has been maintained in the CPqAM insectary since 2007 [8].
4. Resistant field population: Samples of a natural *A. aegypti* population from the municipality of Arcoverde (08°25′08″ S; 37°03′14″ W), 389.7 km from Araripina and 252 km from Recife were previously obtained by collecting eggs using traps (ovitraps), following the procedure of the MoReNAa Network, between July and August 2011 [28]. A sample of this population was kindly provided for this study by the Reference Service for the Control of Culicid Vectors (Serviço de Referência de Controle de Culicídeos Vetores - SRCCV) of the CPqAM- Department of Entomology in 2011. Comparative tests with this population were conducted using the second filial generation (F_2_) to avoid potential influence of maternal and grand-maternal influence.

It is important to highlight that in all the experiments regarding fitness cost described below, RecR, RecRev, RecRNEx and the field population from Arcoverde were all used simultaneously.

### Quantification of the resistance to temephos

In vivo assays were performed to measure the resistance of the larvae of the populations analyzed in this study. In the bioassays, various concentrations of technical grade temephos were used [0.30 to 3.50 mg/mL] (Sigma/97.5 % - batch no. 0535/2011). Tests were performed according to the methodology adapted from the protocol of the World Health Organization [29]. For each concentration of temephos and control, three replicates were used, and at least three independent experiments were performed with each strain to estimate the lethal concentrations (LC) of the insecticide. The resistance ratio was estimated by taking the LC_95_ value of the test strain divided by the observed value for the Rockefeller strain (LC_95_ = 0.011 mg/mL). The resistance degree of the populations were classified according to the criteria established by Mazzarri and Georghiou and were adjusted by the MoReNAa Network into low (3 ≤ RR ≤ 5), medium (5 < RR ≤ 10) or high (RR > 10) resistance [30]. Therefore, samples with a resistance ratio <3 were considered susceptible.

### Quantification of the detoxification enzymes activity

These tests measured the activity of enzymes involved in the detoxification of xenobiotics in *A. aegypti* strains and the field population, previously characterized in relation to their profile of susceptibility to temephos by Araujo et al. [18] and in the present work. The enzymes assessed were mixed-function oxidases (MFOs), glutathione S-transferases (GSTs), and esterases (alpha, beta, and PNPA). Biochemical tests were performed according to the protocol described by the Brazilian Ministry of Health [31]. Approximately 120 unfed females one day post-emergence were analyzed in each group (field population or laboratory strains). At least three independent experiments were performed. The individuals were separately macerated with Milli-Q water (deionized) and homogenized in 1.5 mL microtubes. The homogenates were distributed into 96-well microplates (Nunc®) in duplicate and incubated with their specific substrates. Absorbance readings were performed with a spectrophotometer (Biosystem® *Elx808*) at the proper wavelength for each enzyme. The absorbance results were analyzed using the software *GEN 5*, which transformed the original data (obtained in absorbance values) into enzymatic activity, by calculating the standard deviation of the replicates. The values obtained for each individual were corrected according to the total protein concentration. The enzymatic profiles of the tested groups were classified by comparison with the 99^th^ percentile of the Rockefeller strain. Analyses of the biochemical data classifies populations as unaltered (≤15 %), altered (>15 % and <50 %) and highly altered (>50 %) based on the percentage of individuals from each laboratory strain or field population with enzymatic activity above the Rockefeller 99th percentile [31].

### Biological parameters related to resistance to temephos

#### Investigation of the dynamics of development of the different groups of *Aedes aegypti*

This experiment was performed to assess how long most individuals, with distinct profiles of susceptibility to temephos and raised under the same abiotic conditions (density, pH, light, amount of food and relative humidity), took to reach adulthood. In this test, three replicates (200 L_1_/plastic containers/group) were used in each experiment and three independent experiments were performed, totalizing 1800 L_1_ per group. Larvae were reared according to the conditions described above and the percentage of surviving individuals (larvae, pupae and adults) and the number of males and females (sex ratio) were recorded every three days until the end of the cycle. However, the first record was performed at the 5^th^ day of development.

### Reproductive parameters and longevity of the *Aedes aegypti* females

With the purpose of studying the fecundity, fertility and longevity, groups of 15–20 females were randomly picked from the experiments described above. Newly emerged females from each group of three independent experiments were initially transferred to a containment cage, where they were kept in contact with males for five days before receiving the first blood meal from female Swiss mice (*Mus musculus*) 45 days old. One mouse per group was used. On the day following this procedure, engorged females were carefully placed individually into smaller cages with a cup of water containing filter paper for depositing their eggs. To enhance the mating opportunity, it was also added to each individual cage a male, which remained in contact with the female until his death. Additionally, blood meals were offered to individual female once a week for three consecutive weeks, using different mice. Females that were not fed over the four successive blood meals, as well as those who fed at least once but died during the experiment were excluded from the analyzes. Subsequently, the fecundity (number of eggs per female), fertility (number of L_1_ per number of eggs per female) and longevity (in survival days) were recorded. Likewise, females that did not lay eggs or laid unfertilized eggs were excluded from the analysis of reproductive performance.

### Embryonic viability of eggs with different quiescence times

A set of approximately 200 females, resultant from the experiments of dynamics of population development, were transferred to containment cages and fed weekly with blood to obtain the eggs. Approximately five dried filter papers containing eggs from each group (laboratory strains or field population) were divided into seven parts (with similar quantities of eggs). These papers with eggs were stored in Petri dishes and maintained under controlled conditions (at 26 °C on a 12 h:12 h light:dark cycle at 50–60 % humidity) to evaluate the following quiescence times (Δt): 0, 30, 60, 90, 120, 150, and 180 days, with three replicates for each time point.

### Fertility life table

A fertility life table was constructed based on the methodology described by Silvera Neto and more recently by Diniz et al. to determine the reproductive potential through various variables [32, 33]. The primary variables were: age interval (x), specific fertility (mx) and survival probability (lx). From these variables, the population parameters related to the net reproduction rate (R_O_), generation time (T), intrinsic rate of natural increase (r_m_), finite rate of increase (λ), and time required for the population to double in number of individuals (DT) were calculated, where R_o_ = Σ (l_x_.m_x_), T = Σ (l_x_.m_x_.x)/Σ (l_x_.m_x_), r_m_ = *n* (R_0_)/T, λ = ^erm^ and DT = *n* (2)/r_m_ [32].

### Morphometrics parameters

To estimate the morphometric parameters we used mosquitoes randomly selected from the experiments of dynamics of population development. The wet body weight was estimated from weighing 10 groups of 25 individuals, pupae and unfed adults (males and females), from each strain or population, on an analytical digital high precision scale (BA-002, BEL - Engineering). The size of the females was also estimated by the geometric morphometrics of the wing (right and left) of 15 individuals from each group. The methodology followed was previously described by Monteiro and Reis [34]. The images of the wings, which were mounted between slides and cover slips with Canada balsam, were captured through a photographic camera coupled to a stereomicroscope (Luxeo 4D - Labomed) at 40x magnification. The positional coordinates of 18 anatomical points (landmarks) on a Cartesian plane were taken over the images with the assistance of computer programs (Tpsdig, TpsUtil, and TpsRelw) [35–37]. This data set (related only to size) was used to calculate the centroid size and analyzed by ANOVA test. The data of centroid size and wing shape were associated by canonical variate analysis (CVA), which is a multivariate analysis function used to discriminate different groups [38].

### Quantification of energetic reserves: lipids, glycogen, and other sugars

The contents of lipids, glycogen, and sugars were individually quantified in 50 newly emerged females, randomly selected from the experiment of dynamics of population development using the modified colorimetric technique of Van Handel and Day with a Bio-Rad Smartspec 3000 spectrophotometer for measuring the absorbance data, which were subsequently converted into micrograms of reserve [39, 40]. The energetic value of the sugars and glycogen per individual was calculated by assuming that 1 mg of these carbohydrates is equivalent to 16.74 J and that 1 mg of lipid is equivalent to 37.74 J [41].

### Experimental design and statistical analysis

The experimental design of this study was completely randomized having three replicates in at least three independent experiments. The comparative analyses of the results relating to the tests of susceptibility to temephos of the studied groups (laboratory strains and field population) were calculated through Log-Probit linear regression [42] from the larval mortality observed in the trials after 24 h of exposure to the insecticide using the statistical package SPSS 8.0/Windows. All the tests of fitness cost and quantification of the energetic reserves, comparative analyses were conducted using analysis of variance (ANOVA) and thereafter Tukey’s tests and/or t tests. The data normality was determined using the Shapiro-Wilk test, and the homogeneity variance was tested by Levene’s test. For fecundity, fertility and hatching rate data, normality and homogeneity were achieved by using Log neperiano + mean transformation. The geometric morphometrics of the wing assay were verified by ANOVA (to evaluate the centroid size) and multivariate analysis function (to evaluate the combined shape and size variations). All the analyses were performed using the software Statistic 7.1 (significance level of 5 %). The values obtained for the enzyme activity quantification were statistically analyzed using the software GEN 5*,* which analyzed the absorbance data. These data were transferred to specific Excel spreadsheets standardized by the Brazilian Ministry of Health [31].

## Results and discussion

The results of the present study demonstrate that most biological parameters were compromised in the resistant *Aedes aegypti* strains and field population probably due to the metabolic resistance mechanism.

### Profile of susceptibility to temephos and characterization of the resistance mechanism in the *Aedes aegypti* strains and the field population

The values of the resistance rate (RR) to temephos for the field population and laboratory strains analyzed here were estimated through the LC_95_ values and are shown in Table 1. A peculiarity of our study is that we worked with *A. aegypti* samples with extremely high levels of resistance to temephos (RR > 200). This high-level resistance rate has been observed in recent years in natural populations of this species from Pernambuco and other states in northeast Brazil, but it is rarely reported in the literature [8, 18, 43].

**Table 1:** Profile of susceptibility to temephos for the *Aedes aegypti* laboratory strains and field population

Population	LC_95_^a^ mg/L [CI_95_]	RR_95_^b^	Classification
Rockefeller^c^	0.011 [0.009 - 0.015]	1.0	Susceptible
RecRev ^c^	0.025 [0.018 – 0.039]	2.32	Susceptible
RecRNEx ^c^	2.76 [2.31 – 3.24]	250.5	Resistant
RecR ^c^	3.12 [2.83 – 3.66]	283.6	Resistant
Arcoverde^d,e^	2.44 [2.11–3.00]	222.6	Resistant

^a^LC_95_ = 95 % lethal concentration of temephos; CI = confidence interval

^b^RR_95_ = resistance ratio to the lethal concentration of 95 %. Susceptible (RR < 3), low resistance (3 ≤ RR ≤ 5), moderate resistance (5 < RR ≤ 10) and high resistance (RR > 10)

^c^ laboratory strains; ^d^field population; ^e^data obtained from Araújo et al. (2013)

The RecRNEx strain retained a high level of resistance after five consecutive generations not exposed to temephos. This finding was expected because when the frequency of resistant individuals is very high, the tendency is that the reversal occurs slowly and progressively. Saavedra-Rodriguez et al. selected *A. aegypti* populations from Mexico for five generations using temephos and found an increase in the resistance ratio (RR), which most likely varied according to the initial frequency of resistant individuals. Furthermore, they observed that populations with similar RR values had different levels of resistance after the selective pressure [44]. Nonetheless, both studies show that the evolution of resistance (selection or reversal) depends on the initial frequency of resistant individuals and many other factors, for example, the environmental conditions.

The biochemical assays for characterizing the metabolic resistance mechanism revealed that the enzymatic profile of the laboratory strain used as a control in this study, (RecRev) in its 21^st^ generation, was similar to that found for Rockefeller. Strode et al. also observed this pattern in this same strain at the 13^th^ generation. This fact demonstrates that the detoxification mechanism of the insecticide has receded in RecRev. The enzymatic activity values in all groups studied here are shown in Table 2 [45].

**Table 2:** Profile of enzymatic activities of *Aedes aegypti* groups

Enzyme Class	*Aedes aegypti* Strains/Field Population	p99 ^f^	% > p99 ^g^	Status ^h^
α-esterase (nmol/mg ptn/min)	Rockefeller ^a^	52.99	–	U
	RecRev ^b^		2.0	U
	RecRNEx ^c^		49.0	A
	RecR ^d^		87.0	HA
	Arcoverde ^e^		53.0	A
β–esterase (nmol/mg ptn/min)	Rockefeller	91.98	–	U
	RecRev		1.0	U
	RecRNEx		9.0	U
	RecR		37.0	A
	Arcoverde		4.0	U
PNPA–esterase (Δabs/mg ptn/min)	Rockefeller	4.24	–	U
	RecRev		0.0	U
	RecRNEx		0.0	U
	RecR		57.0	HA
	Arcoverde		18.0	A
MFO nmoles cit/mg ptn)	Rockefeller	9.53	–	U
	RecRev		0.0	U
	RecRNEx		1.0	U
	RecR		5.0	U
	Arcoverde		0.0	U
GST (mmol/mg ptn/min)	Rockefeller	1.46	–	U
	RecRev		9.0	U
	RecRNEx		85.0	HA
	RecR		84.0	HA
	Arcoverde		48.0	A

^a^ susceptible laboratory strain

^b^ susceptible laboratory strain

^c^ resistant laboratory strain (not exposed)

^d^ resistant laboratory strain (exposed)

^e^ resistant field population

^f^ 99^th^ percentile for Rockefeller

^g^ percentage of individuals with a 99^th^ percentile above the 99^th^ percentile for Rockefeller

^h^ classification of enzymatic activity compared to control (Rockefeller): unaltered (U); altered (A); highly altered (HA)

**Fig. 1:**
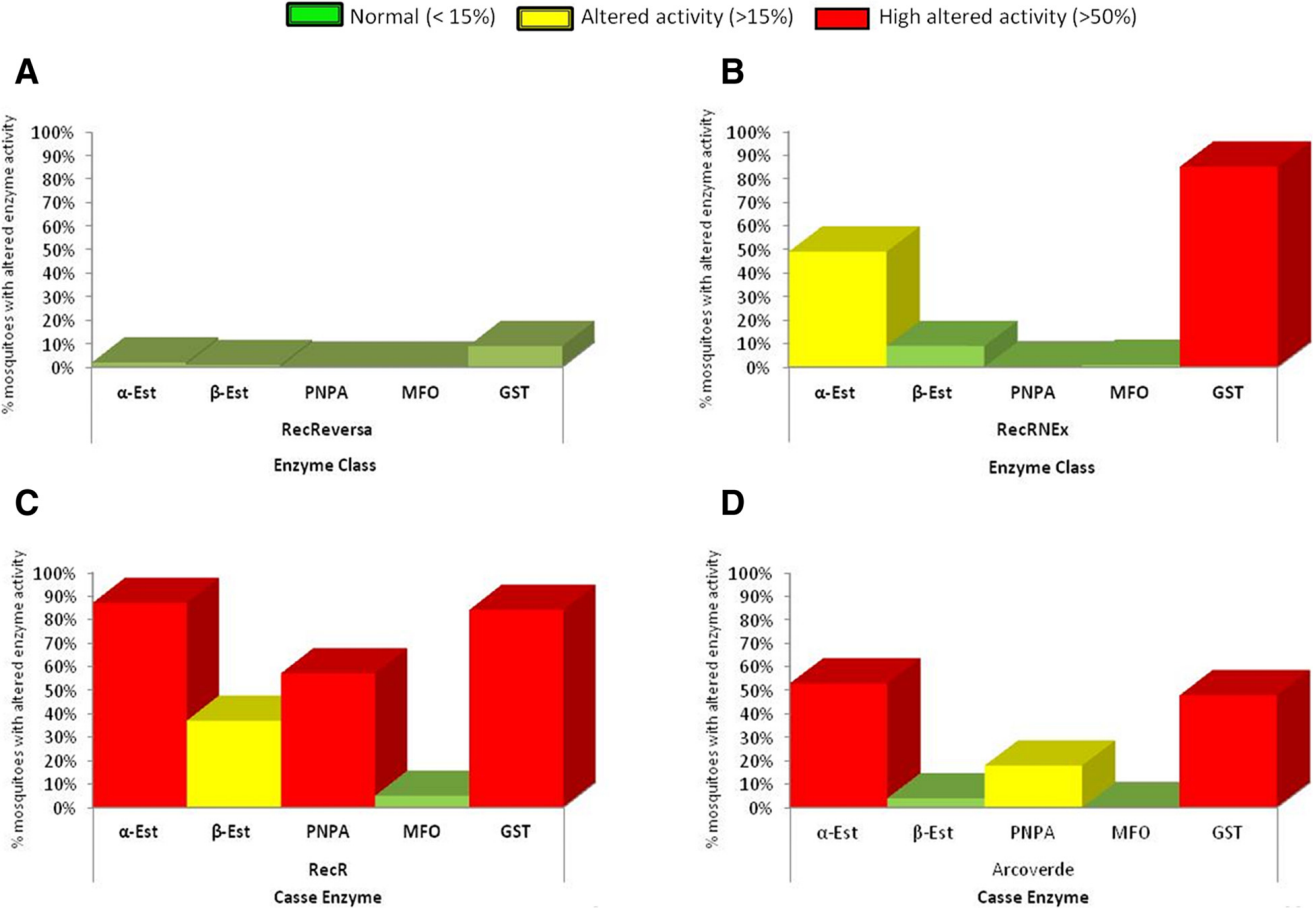
Profile of enzymes related to the detoxification of chemical insecticides in the *Aedes aegypti* laboratory strains and the field population. Alfa-Esterase (α-Est), Beta-Esterase (β-Est), PNPA-esterase (PNPA), Glutathione-S-transferase (GST) and mixed-function oxidases (MFO). The bar indicates the frequency of individuals with altered enzyme activities in the following populations: **a**) RecReverse; **b**) RecRNEx; **c**) RecR and **d**) Arcoverde. The green bar represents population considered normal (when the frequency of mosquitoes with altered enzymatic activity was <15 %), the yellow bar represents population considered altered (when the frequency of mosquitoes with altered enzymatic activities was between 15 and 50 %), and the red bar represents population considered highly altered (frequency of mosquitoes with the altered enzymatic activity >50 %)

The high number of individuals with altered activity of the enzymes α-esterase and GSTs in all resistant strains and the field population confirm the role of this mechanism in mediating resistance to temephos (Fig. 1). The activity of PNPA-esterase was classified as very altered and altered for 60 % and 20 % of 120 individuals analyzed for RecR and Arcoverde, respectively. Only the RecR strain presented changes in β-esterase activity. The profile observed for RecRNEx revealed that the enzymatic activity of PNPA and β-esterase became normal; besides, there was a reduction from 87 % to 49 % of individuals with altered α-esterase activity and maintenance of the altered GST activity (approximately 85 %) after five generations without any contact with temephos. These results suggest that the phenotype of resistance in this strain is primarily associated with GSTs, whose profile remained unchanged, and secondarily with α-esterase enzymes. These findings corroborate previous studies reporting the association between resistance to organophosphates and alterations in the activity of these enzymes in mosquito populations [46–48].

Saavedra-Rodriguez et al. evaluated the transcriptional profile of the metabolic detoxification genes from resistant Mexican *A. aegypti* populations by microarray (Detoxi chip) and verified that the GST Epsilon class enzymes and some esterases display an upregulation pattern compared with the susceptible population [44].

### Dynamics of the population development in the different *Aedes aegypti* groups

The accompaniment of the initial development of larvae of each strain and field population obtained from three independent experiments revealed that for the control group (RecRev), most individuals reached adulthood in 15–18 days after the hatching of L_1_, whereas in resistant strains, an additional 5–7 days of preimaginal development was observed. When evaluating the 5^th^ day of development, a significantly higher number (F = 14.609; df = 3.32, *p* < 0.000005) of 4^th^ instar larvae (L_4_) was observed in the control group compared with the resistant groups. In this assessment, the susceptible strain (RecRev) achieved a mean density of 180.6 (±15.6) larvae, meaning that approximately 90 % of the individuals reached the L_4_ compared with approximately 70 % of RecRNex and 60 % of the resistant mosquitoes from RecR and Arcoverde (Table 3). After 15 days of development, more than 80 % of the individuals of the control group reached adulthood; this value was approximately 60 % for RecRNEx, 45 % for RecR and 26 % for Arcoverde. The larvae and pupae mortality in the control group was only 3.7 %, while in the resistant groups, it ranged from 8.3 % to 13.3 %. Taking the 18^th^ day as a reference, in which 100 % of the surviving individuals reached the adult stage in the control and 80 % in the RecRNEx (*p* = 0.001), a significantly lower number was observed for RecR (*p* < 0.005) and Arcoverde (*p* < 0.05) (F = 6.7383; df = 3.32; *p* = 0.001). Regarding the final number of individuals, no significant differences in the sex ratios (Table 4) were observed among the *A. aegypti* groups studied.

**Table 3:** Larvae and adult densities and cumulative mortality during 18 days of development for different *Aedes aegypti* groups with different patterns of susceptibility to temephos

*Aedes aegypti* groups	Mean L_3_^a^ ± SD Mean L_4_^b^ ± SD ^c^		Mean number of Adults ± SD	Mean Mortality ± SD
	5^th^ day		15^th^ day	5^th^ to 18^th^ day
RecRev	33.7 ± 8.0	180.6 ± 15.6	167.8 ± 22.3	7.4 ± 3.4
RecRNEx	63.2 ± 7.5	134.4 ± 6.6	120.4 ± 10.4	16.5 ± 5.1
RecR	74.3 ± 19.2	116.0 ± 15.4	92.8 ± 14.6	26.6 ± 6.3
Arcoverde	78.5 ± 23.0	115.1 ± 34.4	53.8 ± 28.9	18.4 ± 10.0

The mean represents the amount of larvae, pupae, and adults of nine replicates (with 200 initial larvae)

^a^ L_3_ = 3^rd^ instar larvae

^b ^L_4_ = 4^th^ instar larvae

^c^SD = standard deviation

**Table 4:** The average number of male and female adults obtained from 200 larvae of the different *Aedes aegypti* groups

Population/strains	Male	Female	Sexual rate
	mean ± SD	mean ± SD	mean ± SD
RecRev	95.7 ± 5.3	95.6 ± 6.1	1.06 ± 0.1
RecRNEx	91.0 ± 7.7	92.3 ± 12.0	1.03 ± 0.2
RecR	86.2 ± 11.3	86.7 ± 10.2	1.03 ± 0.3
Arcoverde	89.6 ± 15.1	91.2 ± 16.6	1.07 ± 0.3

In the dynamics of population development, a prolonged larval phase in the natural environment may represent an adaptive disadvantage because individuals would be more exposed to extrinsic risk factors, such as predation, temporary elimination of breeding sites and exposure to xenobiotics, which usually cause a reduced number of generations in the field, as suggested by Berticat et al. [24]. It is important to highlight that *Culex* spp. colonize polluted breeding sites at ground-level in open areas, whereas, the most frequent breeding sites of *A. aegypti* are protected drinking water containers (plastic drum, cement tanks and barrels) [41, 49]. Thus, losses associated with predation and competition in *A. aegypti* are smaller than those in *Culex*. Therefore, in this case, we could speculate that the prolonged larval development time of resistant *A. aegypti* observed in our study might have contributed to individuals making better use of the nutrients available in the rearing plastic containers, partially compensating for the losses associated with maintaining the resistance mechanisms. This can be reproduced under field conditions in real breeding sites of *A. aegypti*, such as in large drink water containers, which are very common in urban environments, especially in areas with precarious water supply system, such as northeast Brazil [50].

On the other hand, another explanation could be that there is some sort of developmental threshold that triggers the beginning of the metamorphosis to the next stage (for instance, accumulation of nutrients), which takes longer for the resistant larvae to reach it, because they expend most of the assimilated resources to maintain resistance, rather than to achieve that threshold. In this case, this would be an adaptative disadvantage.

### Energetic reserves determination in *Aedes aegypti* females

The assessment of the energetic reserves quantification revealed higher lipid concentrations for the individuals of the control group and resistant field population (Arcoverde) compared with those obtained for the resistant lab strains (RecRNEx and RecR) (Table 5). Regarding the glycogen values, a lower concentration of energetic reserves was observed for the RecR strain when compared to the other resistant groups and the control. The concentrations of other less complex sugars (such as glucose and trehalose) were significantly higher in Arcoverde and RecR than in the RecRNEx strain. Table 5 also presents the total energetic values (lipids + carbohydrates) estimated for all the groups analyzed. The highest values were observed for the control group and Arcoverde population. The subtraction of the values of concentrations of glycogen and other sugars resulted in almost no difference between Arcoverde and RecR, and 8 and 7 μg for the control and RecRNEx strain, respectively. These results show that Arcoverde and RecR are probably using the glycogen reserves more than the other groups. This response may be related to the stress caused by continuous contact with the insecticide because the same result was not observed for RecRNEx (unexposed resistant strain). According to Sharma et al. this stress acts as a stimulus to the glycogen catabolism route and the release of other less complex sugars used as energy source in the process of xenobiotic degradation [51].

**Table 5:** Mean amount of energy reserves for lipids and carbohydrates in microgram (μg) and total energetic levels (J), for *Aedes aegypti* females with different patterns of susceptibility to temephos

Population/status of temephos susceptibility	Energy reserves (μg)			Total Energetic values (J)
	Lipids	Glycogen	Other sugars	Lipids + sugars mean ± SD
	mean ± SD	mean ± SD	mean ± SD	
RecRev	71.66 ± 4.94 ^a^	29.53 ± 2.00 ^a^	20.25 ± 1.03 ^c^	3.54 ± 0.8 ^a^
RecRNEx	56.77 ± 4.28 ^b^	24.63 ± 2.46^a^	16.99 ± 1.54 ^d^	2.83 ± 0.3 ^b^
RecR	49.05 ± 3.67^b^	21.88 ± 2.03 ^b^	21.56 ± 2.42 ^bc^	2.58 ± 0.7 ^b^
Arcoverde	70.27 ± 5.39 ^a^	25.05 ± 1.88^a^	24.60 ± 1.45 ^ab^	3.48 ± 0.5 ^a^

Different superscript letters indicate significant differences by Turkey's test (p  < 0,05)

Although the Arcoverde population did not show significant losses in the concentrations of lipid, it did not accumulate considerable gains in its biological potential in relation to other resistant groups. We believe that the reduction of reserves may be related with the production of larger quantities of detoxifying enzymes (especially to alpha-esterase and GSTs), because these enzyme activities were altered in more than 80 % of the individuals of the laboratory strains and 50 % of the field population. Rivero et al. studied organophosphate-resistant *Culex pipiens* strains and reported a significant reduction in the lipid and other sugar reserves when the resistance is caused by the metabolic mechanisms or by target-site alteration [22].

### Morphometric data of *Aedes aegypti*

The size of the pupae and adults were indirectly inferred by the wet body weight and wing size, respectively. Regarding weight, our analysis demonstrated that male pupae (F = 12.35; df = 3.36; *p* = 0.0001) and male adults (F = 6.2918; df = 3.36; *p* = 0.0015) of the control group (RecRev) and Arcoverde population were significantly larger than those of the resistant strains RecRNEx (*p* < 0.0005) and RecR (*p* < 0.0005). Regarding the female pupae, there were no significant differences between the groups studied (Table 6). In contrast, adult females of the control group and Arcoverde were larger (F = 76.30; df = 3.36; *p* < 0.00005) than the females of the resistant strains (*p* < 0.00005).

**Table 6:** Mean weights (g) of pupae and adults in *Aedes aegypti* from three strains and the field population with different pattern of temephos susceptibility

Population/status for temephos susceptibility	Male pupae	Female pupae	Male adults	Female adults
	mean ± SD	mean ± SD	mean ± SD	mean ± SD
RecRev	0.057 ± 0.004 ^a^	0.080 ± 0.020^a^	0.023 ± 0.001^a^	0.053 ± 0.004^a^
RecRNEx	0.049 ± 0.002^b^	0.068 ± 0.006^a^	0.020 ± 0.004^b^	0.030 ± 0.005 ^b^
RecR	0.048 ± 0.005^b^	0.065 ± 0.006^a^	0.018 ± 0.004^b^	0.026 ± 0.005^b^
Arcoverde	0.056 ± 0.004^a^	0.076 ± 0.030^a^	0.024 ± 0.004^a^	0.053 ± 0.006 ^a^

For the purpose of statistical analysis, the data were transformed into the square root of x. Different superscript letters indicate significant differences by Tukey’s test (*p* < 0.05)

**Fig. 2:**
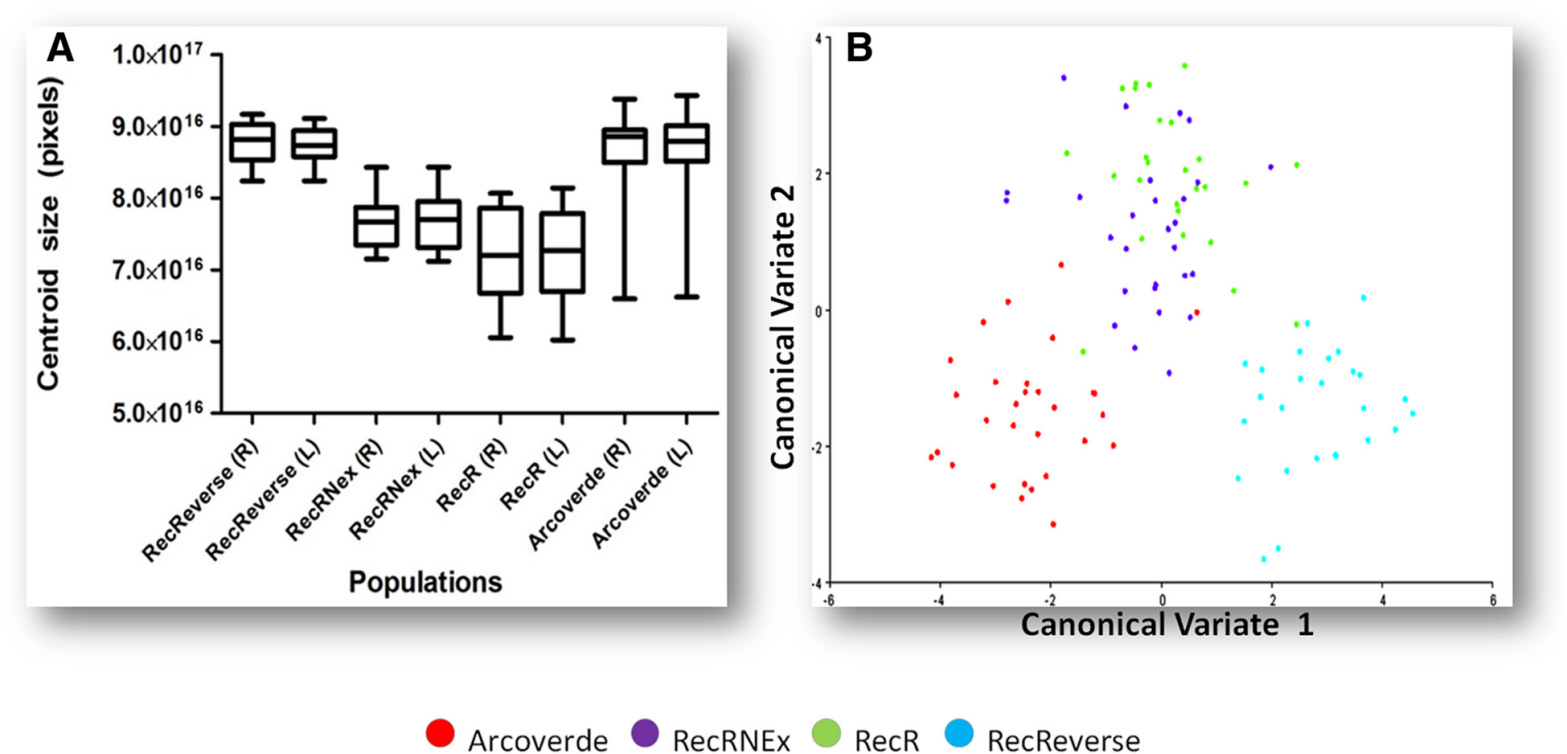
Size and shape analysis of the *Aedes aegypti* females from three strains and the field population through geometric morphometrics of wings. **a**) global isometric sizes (in pixels) of the *A. aegypti* wings with distinctive susceptibility profiles to temephos: RecRev (Susceptible), RecRNEx (resistant non-exposed), RecR (Resistant exposed), and Arcoverde (Resistant field). R: right wing; L: left wing. The central lines show the original means, and the intervals represent the standard error (± SE). **b**) Scatter plot of *A. aegypti* females showing three different clusters: 1) Arcoverde (red); 2) RecRNEx and RecR (purple and green, respectively); and 3) RecRev (blue). Clustering is measured by the canonical variate analysis, which takes into account the shape and size of female wings

In the variate analysis (ANOVA), when we compared the groups regarding the centroid size, we also observed a similarity between the control and the Arcoverde population, and both displayed significant differences (*p* < 0.001) compared to the resistant strains (Fig. 2a). However, when we analyzed the size and shape of the wings of all groups together by canonical variate analysis (CVA), three distinct clusters were observed: 1) RecRev; 2) RecRNEx and RecR and 3) Arcoverde (Fig. 2b). Our results demonstrated that even with isogenetic strains (RecRev, RecRNEx, and RecR), there was a separation into two different clusters. This finding demonstrates that the wing’s geometric morphometric tool was efficient enough to segregate subgroups originated from the same genetic background when they are subjected to different abiotic conditions that can change the pattern of development due to resistance.

Likewise, Jaramillo-O et al. analyzing the effect of resistance to the insecticide lambda-cyhalothrin in *A. aegypti* samples, including a laboratory strain, found no effect on the size of the wings of mosquitoes, only on its shape [52].

Although Arcoverde population has similar levels of resistance of the resistant lab strains, it separated from them, probably because it is a natural field population (with a different genetic background).

### Fecundity, fertility, and longevity of *Aedes aegypti* females

**Fig. 3:**
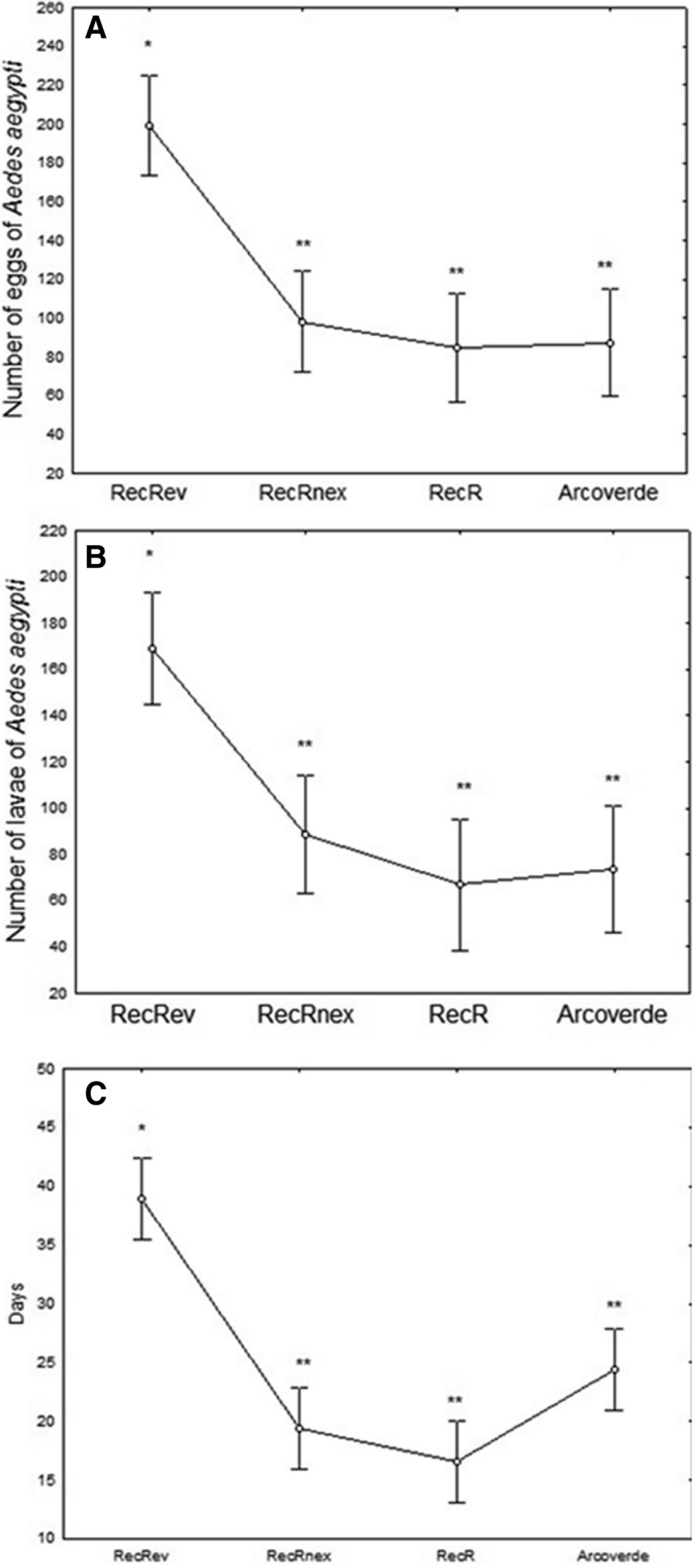
Reproductive parameters and longevity of *Aedes aegypti* females from different strains and the field population. **a**) fecundity (average number of eggs per female); **b**) fertility (average number of L_1/_number of eggs/female) **c**) longevity (average time in days); Columns followed by the same symbol do not differ significantly from each other by Tukey’s test (*p* < 0.0005)

This analysis revealed that the fecundity of resistant females to temephos was reduced to approximately 50 % when compared to the control. Figure 3 presents the data on the fecundity, fertility, and longevity, of the different groups studied. The number of eggs laid per female was significantly higher for the control (RecRev) (F = 93.78; df = 3.164; *p* < 0.000005) compared with all other resistant strains or populations (Fig. 3a), as was the number of viable individuals (L_1_) per female (F = 23.9, df = 3.144; *p* < 0.0005) (Fig. 3b). The average numbers of eggs and L_1_ were, respectively, 205.4 (±125.5) and 175.5 (±107.5) for the control; 107.3 (±70.6) and 89.2 (±66.6) for RecRNEx; 100.6 (±81.5) and 79.0 (±83.0) for Arcoverde; and 93.0 (±49.1) and 64.7 (±40.8) for RecR.

Infertile or non-fecund females were detected in all groups, including the control and the highest percentage was observed in the RecR strain. The cumulative values of these two variables amounted to reductions of 22 %, 32 %, and 40 % of reproductively active females in the RecRNEx, Arcoverde, and RecR strains, respectively, compared with 12 % in the control. These females were excluded from the analysis of fecundity and fertility (Table 7).

**Table 7:** Frequency of reproductive *Aedes aegypti* females with different pattern of temephos susceptibility

Groups	No. females	Non reproductive females		Reproductive females			
		Non fecund	Non fertile	Fertile	Eggs	L_1_	hatching rate (%)
					Mean ± SD	Mean ± SD	
RecRev	50	4	2	44	205.4 ± 125.5	175.5 ± ±107.5	85.4
RecRNEx	50	6	5	39	107.3 ± 70.6	89.2 ± 66.6	83.1
RecR	50	12	8	30	93.0 ± 49.1	64.7 ± 40.8	70.0
Arcoverde	50	10	6	34	100.6 ± ±81.5	79.0 ± 83.0	78.5

The hatching rate was approximately 85 % for RecRev, 83 % for RecRNEx, 78 % for Arcoverde, and 70 % for RecR, with significant differences (F = 21.32; df = 3.14; *p* = 0.005) only between the control group and RecR (*p* = 0.03) and between RecRNEx and RecR (*p* = 0.005) (Table 7). Likewise, Belinato et al. reported differences between susceptible and resistant females to temephos, which produced approximately 20 % fewer eggs (81 ± 30.0) compared with susceptible control females (Rockefeller) (103 ± 19) [53]. Jaramillo-O et al. also observed that *A. aegypti* females resistant to lambda-cyhalothrin were affected in relation to fertility and lifespan [52].

Regarding the data pertaining to the fertility life table (Table 8), the survival rate (lx) and net reproduction rate (R_o_), which corresponds to the number of females generated from each original female, were lower in all resistant groups. The mean duration of one generation (T) was lower for RecR than those observed for all groups. The intrinsic rate of natural increase (r_m_), i.e., the insect’s optimal range of development, demonstrated that for the resistant groups, the growth rate was lower than that observed for the control. The finite rate of increase (λ), which is the number of times the population multiplied per time unit, was also lower for the resistant groups. The generational doubling time (DT) was higher for the RecR and Arcoverde populations than for the other remaining groups. Losses in reproductive potential were confirmed by these variables in the fertility life table, especially the net reproduction rate (Ro), which was twice lower for the resistant females than the control females, followed by a lower intrinsic rate of natural increase (r_m_) as a function of time.

**Table 8:** Fertility life table of *Aedes aegypti* groups with different pattern of temephos susceptibility

Population/status for temephos		Parameters of population growth					
	X (weeks)	l_X_	R_O_	T (weeks)	rm	λ	DT (weeks)
RecRev (susceptible)	4	0.82	45.8	3.5	1.1	3	0.63
RecRNEx (resistant non-exposed)	4	0.54	21.9	3.1	0.99	2.7	0.69
RecR (resistant exposed)	4	0.38	15.8	2.9	0.95	2.6	0.72
Arcoverde (resistant field)	4	0.65	17.4	3.2	0.89	2.4	0.77

X = age interval of females; lx = survival rate during stage x; Ro = net reproductive rate; T = mean duration of each generation; r_m_ = intrinsic rate of natural increase; λ = finite rate of increase; DT = doubling time or the period required for a population to double in size

Diniz et al. also reported losses in the net reproductive rate (Ro) and other variables of the fertility life table, such as the generation time (T), for *A. aegypti* populations from Campina Grande, Paraíba State, Brazil. Still, the Ro values, for example, ranged from 35.5 to 130.7 and were higher than those found in our study, suggesting that the reproductive potential of resistant females from these populations is higher than that observed for all the females of our study [33].

These greater losses in reproductive potential can be justified by the very high levels of resistance (RR > 200) and the presence of the metabolic mechanism mediating this process. Other studies evaluating strains of *Anopheles stephensi* resistant to temephos and propoxur, and *A. aegypti* resistant to pyrethroids, via two different resistance mechanisms, one linked to a knockdown mutation (kdr) and another to a change in the detoxification enzymes activity (α-EST, PNPA-EST, and GST) revealed impairments in the reproductive potential of these species, similar to those described in our study [54, 55].

Regarding longevity, it was observed that females from the control survived an average of 39 (±18) days, with an intragroup variation from 11 to 64 days, while the resistant females survived a significantly shorter time (F = 31.51; df = 3.196; *p* < 0.0005) (Fig. 3c). A reduced longevity for resistant *A. aegypti* was also observed by Belinato et al. who studied other Brazilian *A. aegypti* populations from Boa Vista/Roraima State and Aparecida de Goiâna/Goiás State [53].

Interestingly, the parameters such as size (wing morphology and body weight), amount of energy reserves (lipid and glycogen), and longevity were similar between the control and Arcoverde females. That profile observed for the field population with a high level of resistance to temephos could be explained if we consider that the extension of larval development time may lead to an accumulation of nutrient reserves. Despite this finding, costs were detected in several reproductive parameters for this population compared with the control strain, including a higher percentage of mating females that fed on blood but did not produce eggs or produced them in small amounts.

Assuming that all females have mated, our result suggests that the nutrients obtained during the blood meal were used for maintaining other processes linked to the survival of females instead of egg production. This impairment was confirmed when the variables of the fertility life table (Ro, rm, and λ) revealed losses in the reproductive potential in resistant females to maintain other processes linked to their survival, especially for the laboratory strains. Therefore, the negative pleiotropic effects of resistance to temephos were more evident in our study for the isogenetic strains RecR and RecRNEx than for the Arcoverde field population, although all groups have a similar level of resistance to this compound (RR > 200).

### Viability of eggs with different quiescence times

**Fig. 4:**
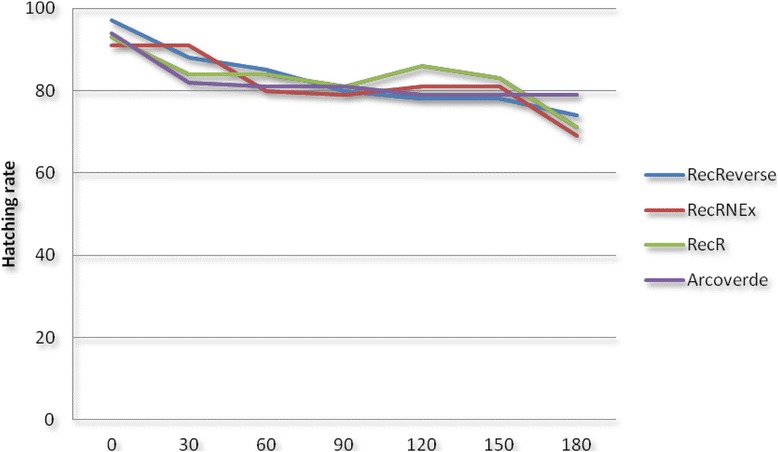
Percent of egg viability of *Aedes aegypti* strains and the field population according to the quiescence time (0–180 days). The lines represent the hatching rate (%) in eggs with different times of quiescence: 0 days; 30 days; 60 days; 90 days; 120 days; 150 days; and 180 days. There was no statistically significant difference between groups

The viability of embryonated eggs at Δt = 0 was approximately 90 % for all the studied groups (strains and field population). After 150 days, the percent viability was still approximately 80 %. At the last evaluation time point (180 days), the hatching rate was higher than 70 % (Fig. 4), with no significant differences in the pattern of larval hatchability among all the groups analyzed. This result confirms the findings by Silva and Silva, who observed viable *A. aegypti* eggs with prolonged quiescence times, showing that this is a problem for public health because eggs are the main form of dispersal in this species (passive dispersion), which hamper its control [56]. Embryonic viability did not decrease in the strains or population evaluated in our study, regardless of their status of susceptibility or resistance to temephos. This aspect is of great importance because the eggs of resistant females, which are quiescent for up to 180 days, were as viable as the susceptible eggs. This undoubtedly contributes to maintaining resistant individuals in the field and helps to explain why the resistance level to temephos in locations where its use has been suspended for more than five years still remains high [9, 18]. According to Paris et al., the dynamics of resistance in the field is highly dependent on the pressure of exposure to the insecticide and on the fitness cost associated with maintaining the mechanism that supports the resistance [57].

In summary, our results confirm the importance of the environmental context, particularly the exposure of populations to adverse conditions that tend to influence survival responses. The results of the present study revealed that 15 of the 19 parameters evaluated changed significantly in the resistant strain RecR and 13 parameters changed for RecRNEx and Arcoverde (resistant field population). Comparing all groups, we found that most of the parameters investigated were negatively affected and nine of them were changed for all resistant groups compared with the control, RecRev (Table 9).

**Table 9:** Summary of fitness cost related to temephos resistance in *Aedes aegypti* laboratory and field populations

Analyzed parameters	RecRev^a^	RecRNEx	RecR	Arcoverde
Mean larval development time (days)	7.5 ± 2.4	=	=	>
Mean egg-adult development time (days)	11.2 ± 5.2	=	=	>
Time (min - max) for obtaining adults (days)	11 to 18	>	>	>
Mortality at the juvenile stage (larvae and pupae)	3.7 %	>	>	>
Size of the females - morphometrics of the wings (pixels)	9.0 × 10^16^	<	<	=
Total energy value/female (lipids and carbohydrates) (J)	3.5	<	<	=
Lipid reserve (μg)	71.66 ± 4.94	<	<	=
Fecundity (eggs/female)	205.4 ± 120	<	<	<
Fertility (L_1_/eggs/female)	175.5 ± 120	<	<	<
Ro (net reproduction rate)	45.8	<	<	<
T (generation time)	3.5	<	<	<
rm (intrinsic rate of natural increase)	1.1	=	<	<
λ (finite rate of increase)	3.0	<	<	<
DT (doubling time of individuals)	0.63	>	>	>
Egg viability (180 days of quiescence)	>70 %	=	=	=
Sex ratio (male/female)	1/1	=	=	=
Reproductive inviability (group of 50 females)	12.0 %	>	>	>
Female longevity (days)	39 ± 18.0	<	<	=
Activity of metabolic enzymes	Unaltered	Altered	Altered	Altered
Number of parameters ≠ of RecRev	NA	13	15	13

RecRev (susceptible), RecRNEx (resistant non-exposed), RecR (resistant exposed), and Arcoverde (resistant field). ^a^ Reference susceptible laboratory population used in the fitness cost tests. The mean values were plotted for each variable studied; NA - not applicable

## Conclusions

Our study revealed that there are fitness costs for *A. aegypti* associated with its resistance to temephos. Furthermore, it was clear that the adaptive disadvantages in populations resistant to this organophosphate, which are caused by the accumulation of negative pleiotropic effects, were particularly reflected in the reproductive parameters, especially when the individuals were selected in the laboratory. Despite this finding, since there is no loss of embryonic viability in the quiescent eggs, resistant individuals would have the same survivability as the susceptible individuals in the field. This trait (prolonged viability) may be critical in promoting the maintenance of residual frequencies of resistant individuals in the field, thereby hindering the effectiveness of management actions.

The resistance to temephos may also promote losses of energetic reserves, particularly in lipids, that are important for physiological activities with a high energetic demand, such as flight, metamorphosis, and egg production. A possible compensation mechanism for these losses observed in our study was the extension of larval development in resistant individuals, a strategy that can minimize deficits related to the survival and reproduction of the females. Moreover, resistance to temephos in *A. aegypti* populations can be reduced or even reversed in the absence of exposure to the insecticide; furthermore, the normal pattern of activity of detoxifying enzymes can be regained. It is important to remember that temephos is still the most widely used larvicide for controlling *A. aegypti* larvae worldwide, although resistance to this compound has been described in various locations, including in non-target species [58].

The data obtained in our study can be considered in the construction of a model of *A. aegypti* population dynamics to estimate whether negative pleiotropic effects of temephos resistance have an impact on the establishment of these populations in the field. In addition, our data may be useful to predict mosquito population trends in areas where insecticide resistance has been detected and resistance management is required.
